# Bufotenidinium iodide

**DOI:** 10.1107/S2414314621001231

**Published:** 2021-02-05

**Authors:** Duyen N. K. Pham, Andrew R. Chadeayne, James A. Golen, David R. Manke

**Affiliations:** a University of Massachusetts Dartmouth, 285 Old Westport Road, North Dartmouth, MA 02747, USA; b CaaMTech, Inc., 58 East Sunset Way, Suite 209, Issaquah, WA 98027, USA; Goethe-Universität Frankfurt, Germany

**Keywords:** crystal structure, natural product, tryptamines, indoles, hydrogen bonding

## Abstract

The title compound, 5-hy­droxy-*N*,*N*,*N*-tri­methyl­tryptammonium (5-HTQ) iodide {systematic name: [2-(5-hy­droxy-1*H*-indol-3-yl)eth­yl]tri­methyl­aza­nium iodide}, C_13_H_19_N_2_O^+^·I^−^, has a single tryptammonium cation and one iodide anion in the asymmetric unit. The ions are held together by N—H⋯I and O—H⋯I hydrogen bonds in infinite chains along [100].

## Abstract 2

The structure of the natural product bufotenidine is reported as its iodide salt.

## Graphical abstract

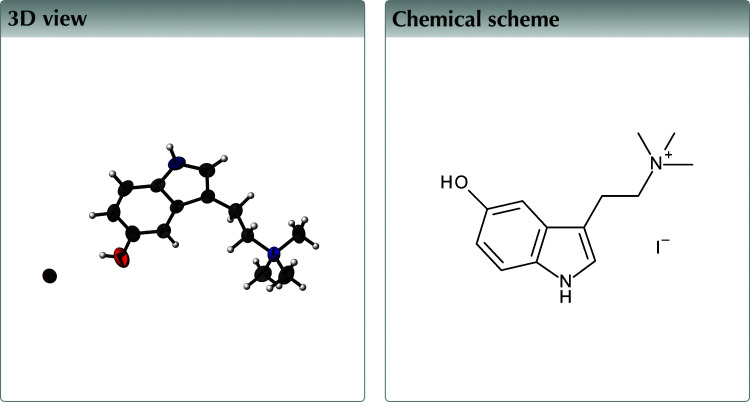

## Structure description

Bufotenidine, the *N*,*N*,*N*-trimethyl analog of serotonin, was first identified in toad secretions in 1934 (Wieland *et al.*, 1934 ▸). This is one of many indo­alkyl­amines found in the secretions of the Colorado River toad, including bufotenine (5-hy­droxy-*N*,*N*-di­methyl­tryptamine), 5-MeO-DMT (5-meth­oxy-*N*,*N*-di­methyl­tryptamine), 5-meth­oxy­tryptophol, and bufoviridine. The primary psychedelic in these secretions, 5-MeO-DMT, has been studied individually to treat anxiety and depression (Davis *et al.*, 2019 ▸). The inhalation of vaporized dried toad secretions has also been examined in the treatment of depression, anxiety and stress (Uthaug *et al.*, 2019 ▸). As this area of research continues, it will be important to understand the difference between pure 5-MeO-DMT and natural toad secretions, to understand the significance of each component, and examine if an entour­age effect is present (Bauer, 2020 ▸). To this end, we have begun to examine some of the minor components of these secretions, and report the first single-crystal structure of the natural product bufotenidine herein.

**Figure 1:**
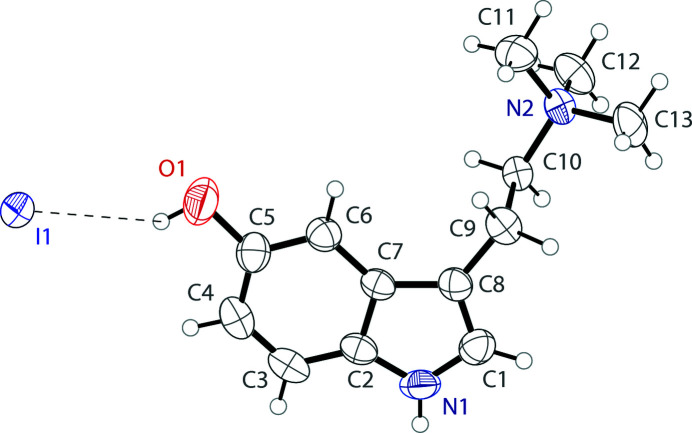
The mol­ecular structure of 5-hy­droxy-*N*,*N*,*N*-tri­methyl­tryptammonium (5-HTQ) iodide, showing the atom labeling. Displacement ellipsoids are drawn at the 50% probability level. A hydrogen bond is shown as a dashed line.

**Figure 2:**
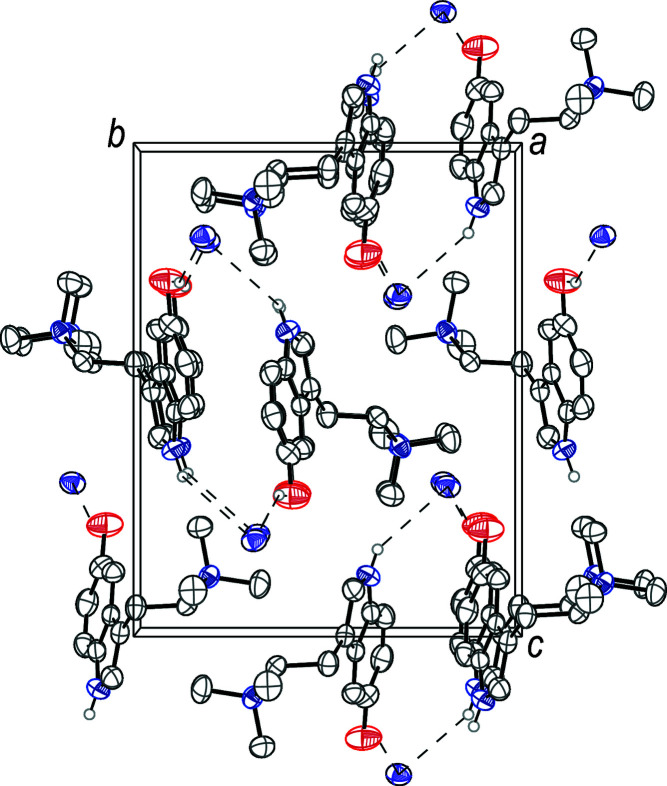
The crystal packing of 5-hy­droxy-*N*,*N*,*N*-tri­methyl­tryptammonium (5-HTQ) iodide, viewed along the *a* axis. The hydrogen bonds (Table 1 ▸) are shown as dashed lines. Displacement ellipsoids are drawn at the 50% probability level. Hydrogen atoms not involved in hydrogen bonds are omitted for clarity.

In the solid-state structure of bufotenidine iodide, the 5-hy­droxy-*N*,*N*,*N*-tri­methyl­tryptammonium cation and the iodide anion are held together in the asymmetric unit *via* O—H⋯I hydrogen bonds (Fig. 1 ▸). The cation possesses a near planar indole group with a mean deviation from planarity of 0.010 Å. The ethyl­amino group is turned away from the plane with a C1—C8—C9—C10 torsion angle of 92.6 (3)°. The N—H of the indole ring hydrogen bonds with a symmetry generated iodide. The N—H⋯I and O—H⋯I hydrogen bonds (Table 1 ▸) link the ions together in infinite chains along the [100] direction with graph-set notation 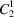 (9) (Etter *et al.*, 1990 ▸). The packing of 5-HTQ iodide is shown in Fig. 2 ▸.

The structure of the closely related natural product from toad secretions, bufotenine, 5-hy­droxy-*N*,*N*-di­methyl­trypt­amine (BUFTEN: Falkenberg, 1972 ▸) has been previously reported. There are only six reported structures of quaternary tryptamines, which are all from the past year. Those are the iodide salts of 4-hy­droxy-*N*,*N*,*N*-tri­methyl­tryptamine (4-HO-TMT) and 4-acet­oxy-*N*,*N*,*N*-tri­methyl­tryptamine (4-AcO-TMT) (XUXFAA and XUXDUS; Chadeayne, Pham, Reid *et al.*, 2020 ▸), *N*,*N*-dimethyl-*N*-*n*-propyl­tryptamine (DMPT) and *N*,*N*-dimethyl-*N*-allyl­tryptamine (DMALT) (CCDC 2017817 and CCDC 2017818; Chadeayne, Pham, Golen & Manke, 2020 ▸), 5-meth­oxy-2-methyl-*N*,*N*,*N*-tri­methyl­tryptamine (5-MeO-2-Me-TMT) and its hydrate (CCDC 2058144 and CCDC 2058145; Pham *et al.*, 2021 ▸).

## Synthesis and crystallization

5-Hy­droxy-*N*,*N*,*N*-tri­methyl­tryptammonium iodide was prepared according to literature procedure (Adhikari *et al.*, 2015 ▸), and crystals suitable for diffraction study were grown from the evaporation of a methanol solution.

## Refinement

Crystal data, data collection and structure refinement details are summarized in Table 2 ▸.

## Supplementary Material

Crystal structure: contains datablock(s) I. DOI: 10.1107/S2414314621001231/bt4107sup1.cif

Structure factors: contains datablock(s) I. DOI: 10.1107/S2414314621001231/bt4107Isup2.hkl

Supporting information file. DOI: 10.1107/S2414314621001231/bt4107Isup3.cml

CCDC reference: 2060560

Additional supporting information:  crystallographic information; 3D view; checkCIF report

## Figures and Tables

**Table 1:** Hydrogen-bond geometry (Å, °)

*D*—H⋯*A*	*D*—H	H⋯*A*	*D*⋯*A*	*D*—H⋯*A*
N1—H1*A*⋯I1^i^	0.85 (1)	2.89 (2)	3.662 (2)	152 (3)
O1—H1⋯I1	0.85 (1)	2.72 (3)	3.468 (2)	147 (4)

Symmetry code: (i) 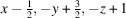 .

**Table 2:** Experimental details

Crystal data	
Chemical formula	C_13_H_19_N_2_O^+^·I^−^
*M*_r_	346.20
Crystal system, space group	Orthorhombic, *P*2_1_2_1_2_1_
Temperature (K)	297
*a*, *b*, *c* (Å)	8.9944 (4), 11.3250 (6), 14.4042 (7)
*V* (Å^3^)	1467.23 (12)
*Z*	4
Radiation type	Mo *K*α
μ (mm^−1^)	2.17
Crystal size (mm)	0.25 × 0.19 × 0.18

Data collection	
Diffractometer	Bruker D8 Venture CMOS
Absorption correction	Multi-scan (*SADABS*; Bruker, 2018 ▸)
*T*_min_, *T*_max_	0.672, 0.745
No. of measured, independent and observed [*I* > 2σ(*I*)] reflections	45268, 2937, 2866
*R*_int_	0.023
(sin θ/λ)_max_ (Å^−1^)	0.625

Refinement	
*R*[*F* ^2^ > 2σ(*F* ^2^)], *wR*(*F* ^2^), *S*	0.013, 0.032, 1.08
No. of reflections	2937
No. of parameters	165
No. of restraints	2
H-atom treatment	H atoms treated by a mixture of independent and constrained refinement
Δρ_max_, Δρ_min_ (e Å^−3^)	0.42, −0.37
Absolute structure	Flack *x* determined using 1216 quotients [(*I* ^+^)−(*I* ^−^)]/[(*I* ^+^)+(*I* ^−^)] (Parsons *et al.*, 2013 ▸)
Absolute structure parameter	−0.021 (4)

Computer programs: *APEX3* and *SAINT* (Bruker, 2018 ▸), *SHELXT2014* (Sheldrick, 2015*b* ▸), *SHELXL2018* (Sheldrick, 2015*a* ▸), *OLEX2* (Dolomanov *et al.*, 2009 ▸) and *publCIF* (Westrip, 2010 ▸).

## full crystallographic data

### Crystal data

**Table:**

C_13_H_19_N_2_O^+^·I^−^	*D*_x_ = 1.567 Mg m^−^^3^
*M**_r_* = 346.20	Mo *K*α radiation, λ = 0.71073 Å
Orthorhombic, *P*2_1_2_1_2_1_	Cell parameters from 9674 reflections
*a* = 8.9944 (4) Å	θ = 2.7–26.4°
*b* = 11.3250 (6) Å	µ = 2.17 mm^−^^1^
*c* = 14.4042 (7) Å	*T* = 297 K
*V* = 1467.23 (12) Å^3^	BLOCK, colourless
*Z* = 4	0.25 × 0.19 × 0.18 mm
*F*(000) = 688	

### Data collection

**Table:**

Bruker D8 Venture CMOS diffractometer	2866 reflections with *I* > 2σ(*I*)
φ and ω scans	*R*_int_ = 0.023
Absorption correction: multi-scan (SADABS; Bruker, 2018)	θ_max_ = 26.4°, θ_min_ = 2.9°
*T*_min_ = 0.672, *T*_max_ = 0.745	*h* = −11→10
45268 measured reflections	*k* = −14→14
2937 independent reflections	*l* = −18→18

### Refinement

**Table:**

Refinement on *F*^2^	Primary atom site location: dual
Least-squares matrix: full	Hydrogen site location: mixed
*R*[*F*^2^ > 2σ(*F*^2^)] = 0.013	H atoms treated by a mixture of independent and constrained refinement
*wR*(*F*^2^) = 0.032	*w* = 1/[σ^2^(*F*_o_^2^) + (0.0105*P*)^2^ + 0.4198*P*] where *P* = (*F*_o_^2^ + 2*F*_c_^2^)/3
*S* = 1.08	(Δ/σ)_max_ = 0.002
2937 reflections	Δρ_max_ = 0.42 e Å^−^^3^
165 parameters	Δρ_min_ = −0.37 e Å^−^^3^
2 restraints	

### Special details

**Table:**

Geometry. All esds (except the esd in the dihedral angle between two l.s. planes) are estimated using the full covariance matrix. The cell esds are taken into account individually in the estimation of esds in distances, angles and torsion angles; correlations between esds in cell parameters are only used when they are defined by crystal symmetry. An approximate (isotropic) treatment of cell esds is used for estimating esds involving l.s. planes.
Refinement. The H atoms bonded to C were positioned geometrically and refined using a riding model with C—H = 0.97 Å and *U*_iso_(H) = 1.2*U*eq(C) for CH_2_ and C—H = 0.93 Å and *U*_iso_(H) = 1.2*U*eq(C) for CH and with C—H = 0.96 Å and *U*_iso_(H) = 1.5*U*eq(C) for CH_3_. The H atoms bonded to O and N were refined isotropically with a distance restraint of 0.86 (1) Å for O—H and N—H.

### Fractional atomic coordinates and isotropic or equivalent isotropic displacement parameters (Å^2^)

**Table:**

	*x*	*y*	*z*	*U*_iso_*/*U*_eq_	
I1	1.27678 (2)	0.68783 (2)	0.80214 (2)	0.04918 (6)	
O1	0.9362 (3)	0.5938 (3)	0.71675 (17)	0.0750 (7)	
N1	0.6590 (3)	0.6055 (2)	0.37895 (16)	0.0498 (5)	
N2	0.2789 (2)	0.30817 (18)	0.61774 (13)	0.0391 (4)	
C1	0.5215 (3)	0.5666 (2)	0.40501 (19)	0.0485 (6)	
H1B	0.441446	0.556178	0.364948	0.058*	
C2	0.7484 (3)	0.61161 (18)	0.45606 (16)	0.0406 (6)	
C3	0.8962 (3)	0.6469 (2)	0.4660 (2)	0.0497 (7)	
H3	0.950871	0.673197	0.415302	0.060*	
C4	0.9584 (3)	0.6418 (2)	0.5530 (2)	0.0507 (7)	
H4	1.056532	0.665360	0.561501	0.061*	
C5	0.8756 (3)	0.6014 (2)	0.6292 (2)	0.0479 (6)	
C6	0.7298 (3)	0.5668 (2)	0.62022 (16)	0.0429 (5)	
H6	0.676325	0.540356	0.671410	0.052*	
C7	0.6637 (3)	0.57222 (19)	0.53238 (16)	0.0364 (5)	
C8	0.5182 (3)	0.5451 (2)	0.49817 (17)	0.0391 (5)	
C9	0.3882 (3)	0.5012 (2)	0.55239 (19)	0.0447 (6)	
H9A	0.296470	0.525310	0.522426	0.054*	
H9B	0.389880	0.534545	0.614414	0.054*	
C10	0.3953 (3)	0.3676 (2)	0.55785 (17)	0.0358 (5)	
H10A	0.386906	0.336400	0.495364	0.043*	
H10B	0.492611	0.345643	0.581186	0.043*	
C11	0.3074 (4)	0.3325 (3)	0.71866 (17)	0.0547 (7)	
H11A	0.406203	0.307458	0.734450	0.082*	
H11B	0.236688	0.290083	0.755761	0.082*	
H11C	0.297675	0.415645	0.730285	0.082*	
C12	0.2907 (3)	0.1772 (2)	0.6019 (2)	0.0566 (6)	
H12A	0.388997	0.151050	0.617656	0.085*	
H12B	0.271215	0.160013	0.537743	0.085*	
H12C	0.219355	0.136997	0.640060	0.085*	
C13	0.1260 (3)	0.3483 (3)	0.5916 (2)	0.0599 (8)	
H13A	0.113955	0.429832	0.608060	0.090*	
H13B	0.053621	0.301558	0.624009	0.090*	
H13C	0.112305	0.339099	0.525871	0.090*	
H1A	0.686 (4)	0.629 (3)	0.3256 (13)	0.070 (10)*	
H1	1.021 (2)	0.625 (3)	0.713 (3)	0.093 (14)*	

### Atomic displacement parameters (Å^2^)

**Table:**

	*U*^11^	*U*^22^	*U*^33^	*U*^12^	*U*^13^	*U*^23^
I1	0.04798 (9)	0.05760 (10)	0.04197 (8)	−0.00650 (8)	−0.00644 (7)	0.00349 (8)
O1	0.0540 (13)	0.118 (2)	0.0527 (13)	−0.0119 (14)	−0.0117 (10)	−0.0055 (13)
N1	0.0669 (15)	0.0466 (12)	0.0357 (11)	−0.0056 (11)	0.0020 (11)	0.0092 (9)
N2	0.0353 (8)	0.0375 (9)	0.0444 (9)	−0.0029 (11)	0.0023 (8)	−0.0056 (8)
C1	0.0556 (15)	0.0419 (13)	0.0480 (14)	0.0001 (12)	−0.0082 (12)	0.0017 (12)
C2	0.0484 (17)	0.0282 (10)	0.0450 (13)	−0.0017 (10)	0.0060 (11)	0.0012 (9)
C3	0.0529 (16)	0.0398 (13)	0.0564 (16)	−0.0067 (12)	0.0170 (14)	0.0026 (12)
C4	0.0401 (14)	0.0433 (13)	0.0688 (18)	−0.0059 (11)	0.0041 (13)	−0.0079 (13)
C5	0.0463 (15)	0.0500 (15)	0.0473 (14)	−0.0004 (12)	−0.0031 (12)	−0.0097 (11)
C6	0.0443 (12)	0.0455 (12)	0.0389 (11)	−0.0029 (12)	0.0044 (11)	−0.0020 (9)
C7	0.0432 (12)	0.0263 (10)	0.0396 (12)	−0.0014 (9)	0.0050 (10)	−0.0021 (9)
C8	0.0441 (13)	0.0288 (11)	0.0442 (13)	−0.0010 (10)	−0.0002 (11)	−0.0021 (10)
C9	0.0402 (13)	0.0375 (13)	0.0564 (16)	0.0024 (11)	0.0048 (12)	−0.0018 (12)
C10	0.0325 (11)	0.0365 (11)	0.0385 (12)	−0.0011 (9)	0.0010 (9)	−0.0048 (9)
C11	0.0672 (19)	0.0554 (16)	0.0414 (13)	−0.0034 (13)	0.0083 (11)	−0.0036 (11)
C12	0.0596 (15)	0.0380 (12)	0.0721 (17)	−0.0109 (14)	0.0107 (14)	−0.0071 (13)
C13	0.0313 (12)	0.069 (2)	0.080 (2)	−0.0019 (12)	0.0037 (14)	0.0006 (16)

### Geometric parameters (Å, º)

**Table:**

O1—C5	1.377 (4)	C6—H6	0.9300
O1—H1	0.847 (13)	C6—C7	1.399 (3)
N1—C1	1.366 (4)	C7—C8	1.432 (3)
N1—C2	1.373 (3)	C8—C9	1.492 (4)
N1—H1A	0.849 (13)	C9—H9A	0.9700
N2—C10	1.515 (3)	C9—H9B	0.9700
N2—C11	1.502 (3)	C9—C10	1.516 (3)
N2—C12	1.504 (3)	C10—H10A	0.9700
N2—C13	1.496 (3)	C10—H10B	0.9700
C1—H1B	0.9300	C11—H11A	0.9600
C1—C8	1.364 (4)	C11—H11B	0.9600
C2—C3	1.395 (4)	C11—H11C	0.9600
C2—C7	1.410 (3)	C12—H12A	0.9600
C3—H3	0.9300	C12—H12B	0.9600
C3—C4	1.373 (4)	C12—H12C	0.9600
C4—H4	0.9300	C13—H13A	0.9600
C4—C5	1.403 (4)	C13—H13B	0.9600
C5—C6	1.374 (4)	C13—H13C	0.9600
			
C5—O1—H1	106 (3)	C1—C8—C9	126.2 (3)
C1—N1—C2	108.9 (2)	C7—C8—C9	127.4 (2)
C1—N1—H1A	128 (2)	C8—C9—H9A	109.9
C2—N1—H1A	123 (2)	C8—C9—H9B	109.9
C11—N2—C10	110.56 (19)	C8—C9—C10	109.0 (2)
C11—N2—C12	108.4 (2)	H9A—C9—H9B	108.3
C12—N2—C10	107.63 (18)	C10—C9—H9A	109.9
C13—N2—C10	110.9 (2)	C10—C9—H9B	109.9
C13—N2—C11	110.2 (2)	N2—C10—C9	116.3 (2)
C13—N2—C12	109.0 (2)	N2—C10—H10A	108.2
N1—C1—H1B	124.8	N2—C10—H10B	108.2
C8—C1—N1	110.3 (2)	C9—C10—H10A	108.2
C8—C1—H1B	124.8	C9—C10—H10B	108.2
N1—C2—C3	131.0 (2)	H10A—C10—H10B	107.4
N1—C2—C7	107.4 (2)	N2—C11—H11A	109.5
C3—C2—C7	121.7 (2)	N2—C11—H11B	109.5
C2—C3—H3	121.0	N2—C11—H11C	109.5
C4—C3—C2	118.1 (3)	H11A—C11—H11B	109.5
C4—C3—H3	121.0	H11A—C11—H11C	109.5
C3—C4—H4	119.7	H11B—C11—H11C	109.5
C3—C4—C5	120.7 (3)	N2—C12—H12A	109.5
C5—C4—H4	119.7	N2—C12—H12B	109.5
O1—C5—C4	121.7 (3)	N2—C12—H12C	109.5
C6—C5—O1	116.5 (3)	H12A—C12—H12B	109.5
C6—C5—C4	121.8 (3)	H12A—C12—H12C	109.5
C5—C6—H6	120.7	H12B—C12—H12C	109.5
C5—C6—C7	118.5 (2)	N2—C13—H13A	109.5
C7—C6—H6	120.7	N2—C13—H13B	109.5
C2—C7—C8	107.1 (2)	N2—C13—H13C	109.5
C6—C7—C2	119.3 (2)	H13A—C13—H13B	109.5
C6—C7—C8	133.6 (2)	H13A—C13—H13C	109.5
C1—C8—C7	106.3 (2)	H13B—C13—H13C	109.5
			
O1—C5—C6—C7	−179.6 (2)	C3—C2—C7—C8	179.1 (2)
N1—C1—C8—C7	−0.1 (3)	C3—C4—C5—O1	179.0 (3)
N1—C1—C8—C9	−179.7 (2)	C3—C4—C5—C6	−0.7 (4)
N1—C2—C3—C4	−178.8 (3)	C4—C5—C6—C7	0.1 (4)
N1—C2—C7—C6	178.5 (2)	C5—C6—C7—C2	0.6 (3)
N1—C2—C7—C8	−1.5 (3)	C5—C6—C7—C8	−179.4 (3)
C1—N1—C2—C3	−179.3 (3)	C6—C7—C8—C1	−179.0 (3)
C1—N1—C2—C7	1.5 (3)	C6—C7—C8—C9	0.6 (4)
C1—C8—C9—C10	92.6 (3)	C7—C2—C3—C4	0.4 (4)
C2—N1—C1—C8	−0.8 (3)	C7—C8—C9—C10	−86.9 (3)
C2—C3—C4—C5	0.4 (4)	C8—C9—C10—N2	174.8 (2)
C2—C7—C8—C1	1.0 (3)	C11—N2—C10—C9	−70.4 (3)
C2—C7—C8—C9	−179.4 (2)	C12—N2—C10—C9	171.4 (2)
C3—C2—C7—C6	−0.9 (3)	C13—N2—C10—C9	52.2 (3)

### Hydrogen-bond geometry (Å, º)

**Table:**

*D*—H···*A*	*D*—H	H···*A*	*D*···*A*	*D*—H···*A*
N1—H1*A*···I1^i^	0.85 (1)	2.89 (2)	3.662 (2)	152 (3)
O1—H1···I1	0.85 (1)	2.72 (3)	3.468 (2)	147 (4)

Symmetry code: (i) *x*−1/2, −*y*+3/2, −*z*+1.

## References

[bb1] Adhikari, B. B., Roshandel, S., Fujii, A. & Schramm, M. P. (2015). *Eur. J. Org. Chem.* **2015**, 2683–2690. 10.1002/ejoc.201403519 PMC4495001 26161035

[bb2] Bauer, B. E. (2020). *Psychedelic Science Review*. https://psychedelicreview.com/hamilton-morris-on-5-meo-dmt-the-entourage-effect-and-protecting-toads/

[bb3] Bruker (2018). *APEX3*, *SAINT*, and *SADABS*. Bruker AXS Inc., Madison, Wisconsin, USA.

[bb4] Chadeayne, A. R., Pham, D. N. K., Golen, J. A. & Manke, D. R. (2020). *Acta Cryst.* E**76**, 1357–1360. 10.1107/S2056989020010014 PMC7405565 32844029

[bb5] Chadeayne, A. R., Pham, D. N. K., Reid, B. G., Golen, J. A. & Manke, D. R. (2020). *ACS Omega*, **5**, 16940–16943. 10.1021/acsomega.0c02208 PMC7365549 32685863

[bb6] Davis, A. K., So, S., Lancelotta, R., Barsuglia, J. P. & Griffiths, R. R. (2019). *Am. J. Drug Alcohol Abuse*, **45**, 161–169. 10.1080/00952990.2018.1545024 PMC6430661 30822141

[bb7] Dolomanov, O. V., Bourhis, L. J., Gildea, R. J., Howard, J. A. K. & Puschmann, H. (2009). *J. Appl. Cryst.* **42**, 339–341.

[bb8] Etter, M. C., MacDonald, J. C. & Bernstein, J. (1990). *Acta Cryst.* B**46**, 256–262. 10.1107/s0108768189012929 2344397

[bb9] Falkenberg, G. (1972). *Acta Cryst.* B**28**, 3219–3228.

[bb10] Parsons, S., Flack, H. D. & Wagner, T. (2013). *Acta Cryst.* B**69**, 249–259. 10.1107/S2052519213010014 PMC3661305 23719469

[bb11] Pham, D. N. K., Chadeayne, A. R., Golen, J. A. & Manke, D. R. (2021). *Acta Cryst.* E**77**, 190–194. 10.1107/S2056989021000803 PMC7869544 33614152

[bb12] Sheldrick, G. M. (2015*a*). *Acta Cryst.* A**71**, 3–8.

[bb13] Sheldrick, G. M. (2015*b*). *Acta Cryst.* C**71**, 3–8.

[bb14] Uthaug, M. V., Lancelotta, R., van Oorsouw, K., Kuypers, K. P. C., Mason, N., Rak, J., Šuláková, A., Jurok, R., Maryška, M., Kuchař, M., Páleníček, T., Riba, J. & Ramaekers, J. G. (2019). *Psychopharmacology*, **236**, 2653–2666. 10.1007/s00213-019-05236-w PMC6695371 30982127

[bb15] Westrip, S. P. (2010). *J. Appl. Cryst.* **43**, 920–925.

[bb16] Wieland, H., Konz, W. & Mittasch, H. (1934). *Justus Liebigs Ann. Chem.* **513**, 1–25.

